# Draft Genome Sequence of *Massilia* sp. Strain MC02, Isolated from a Sandy Loam Maize Soil

**DOI:** 10.1128/MRA.00410-19

**Published:** 2019-08-08

**Authors:** Rachel Raths, Vincent Peta, Heike Bücking

**Affiliations:** a Biology and Microbiology Department, South Dakota State University, Brookings, South Dakota, USA; University of Arizona

## Abstract

From farmed corn soil in California, we isolated and sequenced a new member of the genus Massilia, *Massilia* sp. strain MC02. *Massilia* sp. MC02 has an assembled draft genome of 5,023,356 bp with a total of 4,790 protein-encoding genes and 3,028 predicted proteins, 47 tRNA genes, and 2 rRNA operons.

## ANNOUNCEMENT

The first species within the genus *Massilia* was isolated from clinical samples (1). Since then, *Massilia* species have been isolated from plant tissues (2), water (3), air (4), ice cores (5), and soils (6). *Massilia* spp. have been shown to be abundant in the plant rhizosphere and to colonize roots (7, 8). Some species have plant growth-promoting capabilities, such as the production of indole-3-acetic acid (9) or siderophores, and are involved in soil carbon and nitrogen cycling (10). *Massilia* is the most species-rich genus of the Oxalobacteraceae family and consists mainly of Gram-negative, aerobic, non-spore-forming, motile rods.

*Massilia* sp. strain MC02 was isolated from a maize rhizosphere sample from a sandy loam soil in California on 22 May 2015. The geographical coordinates are 37.6058, −120.7478. Soil was added to phosphate-buffered saline, and dilutions were plated on Reasoner’s 2A (R2A) agar plates and incubated at 30°C for 1 to 2 days, followed by 20°C for 1 to 2 days. Sequential colony streaks were performed on R2A agar to acquire pure colonies. Genomic DNA was extracted from a freshly grown R2A broth culture using the AllPrep bacterial DNA/RNA/protein kit (Qiagen, Inc., Germantown, MD) following the kit protocol. The genomic library was prepared with Illumina Nextera technology (San Diego, CA), size selected to an average fragment length of 475 bp, and sequenced using Illumina NextSeq paired-end v2 chemistry on v2.5 flow cells at 150 bp per read. A target coverage of 20× was used, and the genome was assembled using SPAdes v3.11.0 (11). Default parameters were used for all software unless otherwise specified. We obtained 1,566,408 total reads, with an average read length of 148 bp. The total read length was 231,096,882 bp, with 275 contigs, an *N*_50_ value of 28,267 bp (range, 1,074 to 119,695 bp), and an *L*_50_ value of 49. The genome length was 5,023,356 bp, with a GC content of 66.2%. Assembly quality assessment using BUSCO (12) revealed a measured completeness of 95%. Gene prediction and annotation using PATRIC v3.5.27 (13) resulted in a total of 4,790 protein-coding sequences consisting of 1,762 hypothetical proteins and 3,028 proteins with functional assignments, 47 tRNA genes, and 2 rRNA operons. The Microbial Genomes Atlas (MiGA) (14) revealed that the closest related strain from the NCBI database is Massilia armeniaca ZMN-3 (GenBank accession number NZ_CP028324), with an amino acid identity (AAI) of 67.37%. Based on the MiGA results, MC02 belongs to the *Massilia* genus and was designated *Massilia* sp. strain MC02.

Using Galaxy (15), several genes with putative plant growth-promoting characteristics were identified, such as a nitrate reductase gene (*napA*), several phosphatase genes (*ppk*, *phoA*, *phoB*, *phoD*, and *phoR*), and biotin biosynthesis genes (*bioA*, *bioB*, *bioD*, and *bioF*). Using RAST 2.0 (16), we identified 48 putative virulence genes, including 34 genes indicating a resistance to antibiotics and toxic compounds, 14 genes putatively involved in invasion and intracellular resistance, and 26 genes responsible for flagellar motility. Invasion and flagellum genes are essential for attaching and entering plant cells (17), suggesting that MC02 is a plant endophyte.

### Data availability.

The complete genome sequence has been deposited in NCBI/EBI/GenBank under BioProject number PRJNA529270, BioSample number SAMN11263498, GenBank accession number SPVF00000000, and SRA accession number SRX6098478.
